# Lumbar disc extrusions reduce faster than bulging discs due to an active role of macrophages in sciatica

**DOI:** 10.1007/s00701-019-04117-7

**Published:** 2019-12-04

**Authors:** N. Djuric, X. Yang, A. el Barzouhi, R. Ostelo, S. G. van Duinen, G. J. Lycklama à Nijeholt, B. F. W. van der Kallen, W. C. Peul, C. L. A. Vleggeert-Lankamp

**Affiliations:** 1 Department of Neurosurgery, Leiden University Medical Center, Albinusdreef 2, 2300 RC Leiden, Netherlands; 2 Department of Epidemiology, VU Medical Centre, Amsterdam, Netherlands; 3 Department of Health Sciences, Faculty of Science, Amsterdam Movement Sciences Research Institute, Vrije Universiteit, Amsterdam, Netherlands; 4 Department of Pathology, Leiden University Medical Center, Leiden, Netherlands; 5 Haaglanden Medical Center, The Hague, Netherlands

**Keywords:** Sciatica, Inflammation, Macrophages, Disc herniation, Magnetic resonance imaging, Modic changes

## Abstract

**Objective:**

This retrospective observational histological study aims to associate the size and type of disc herniation with the degree of macrophage infiltration in disc material retrieved during disc surgery in patients with sciatica.

**Methods:**

Disc tissue of 119 sciatica patients was embedded in paraffin and stained with hematoxylin and CD68. Tissue samples were categorized as mild (0–10/cm^2^), moderate (10–100/cm^2^), and considerable (> 100/cm^2^) macrophage infiltration. All 119 patients received an MRI at baseline, and 108 received a follow-up MRI at 1-year. MRIs were reviewed for the size and type of the disc herniations, and for Modic changes in the vertebral endplates.

**Results:**

Baseline characteristics and duration of symptoms before surgery were comparable in all macrophage infiltration groups. The degree of macrophage infiltration was not associated with herniation size at baseline, but significantly associated with reduction of size of the herniated disc at 1-year post surgery. Moreover, the degree of macrophage infiltration was higher in extrusion in comparison with bulging (protrusion) of the disc. Results were comparable in patients with and without Modic changes.

**Conclusion:**

Macrophage infiltration was positively associated with an extruded type of disc herniation as well as the extent of reduction of the herniated disc during 1-year follow-up in patients with sciatica. This is an indication that the macrophages play an active role in reducing herniated discs. An extruded disc herniation has a larger surface for the macrophages to adhere to, which leads to more size reduction.

**Electronic supplementary material:**

The online version of this article (10.1007/s00701-019-04117-7) contains supplementary material, which is available to authorized users.

## Introduction

Herniation of the intervertebral disc is a highly prevalent disease [11], characterized by radiating pain symptoms due to a compression of the nerve root by the herniated disc. Since 1934, it has been widely accepted that a disc may herniate due to a disruption of the annulus fibrosus or due to its attachments to the adjacent endplate [16]. This disruption of the annulus fibrosus can result in a foreign-body reaction aimed at the nucleus pulposus that induces neovascularization [13], which is accompanied by macrophage infiltration [15, 24, 26].

This macrophage infiltration is believed to exacerbate pain symptoms through secretion of pro-inflammatory cytokines such as IL-6, IL8, and TNF-alfa [1, 20, 29]. In contrast, macrophage infiltration may also have a positive effect on symptoms through inducing a phagocytic resorption process, mediated by anti-inflammatory cytokines such as IL-4 and IL-10 [27, 29]. This discrepancy in effect of macrophage infiltration is reflected in the inconsistent findings regarding the correlation between macrophage infiltration and clinical symptoms [14, 23–25, 28]

A possible factor that may reflect the type of macrophage is the presence of Modic changes (MC), also known as vertebral endplate signal changes (VESC) on MRI [17, 18]. Recent studies have shown that patients with MC recover more slowly from their herniated discs than those without MC [2, 26]. In addition, Dudli et al. showed that both Modic type 1 and type 2 changes are associated with inflammatory dysmyelopoiesis and fibrogenic changes [4], which infers that MC reflect a factor that plays a role in the inflammatory process in the disc. Hence, it is to be expected that this factor in the inflammatory process, reflected by presence of MC, interferes with the resorption process, possibly by changing macrophage differentiation away from the anti-inflammatory type and towards the pro-inflammatory type. Until now, the interaction between MC and macrophage infiltration on the resorption of the herniated disc (size reduction) remains unknown.

Furthermore, if a herniated disc extrudes instead of bulges (protrudes), it is likely to have a higher exposure to the systemic circulation through more neovascularization, which in turn may result in more macrophage infiltration. However, the evidence supporting this theory is limited [15]. In addition, recent studies have found that MC are associated with less neovascularization [12, 26]. It is thus hypothesized that the presumed association between macrophage infiltration and disc extrusion will be less strong in patients with MC.

In order to test the previously mentioned theories, this study aims to associate macrophage infiltration with the type and size of disc herniation as well as the reduction in disc herniation at 1-year follow-up in patients with sciatica. We will also explore this association in patient with and without MC separately. Exploring these associations will provide a more profound understanding of the roles of macrophage infiltration and MC in patients with sciatica due to a lumbar disc herniation.

## Materials and methods

### Study population

This retrospective study was performed using participants from the Sciatica Trial [22], a multicenter RCT with 283 patients who suffered from sciatica for 6–12 weeks and had a disc herniation as assessed by means of MRI. A total of 141 patients were randomized to surgery and 125 patients actually underwent surgery (16 recovered before surgery could be performed). The other 142 patients were randomized to prolonged conservative care, of which 55 patients underwent surgery within 1 year, with a mean time to surgery of 15 weeks after randomization. Thus, in the first year after randomization, a total of 180 patients underwent surgery for sciatica. Out of the 180 patients, 120 disc samples were available for analysis. Missing samples were due to multiple reasons: not collected during surgery, got lost after surgery, not preserved properly, or got lost after preservation. All surgeries were performed between November 2002 and February 2005. The protocol, which included analysis of the disc material, was approved by the medical ethics committees at all participating hospitals.

### Histological analysis

Disc material of all operated patients was collected and fixed in 4% formaldehyde solution after surgery and was subsequently stored for future analysis. For the purpose of this retrospective study, samples were embedded in blocks of paraffin and stained with CD68 to evaluate macrophage infiltration. A detailed description of the protocol was published in our previous work [3].

The evaluation was done by two independent investigators, who were blinded to clinical information and MRI data. The training of these independent researchers was carried out by a senior pathologist. The number of macrophages on each sample was counted and estimated. Using this method, the tissue samples were categorized based on the extent of macrophage infiltration. The categories consisted of mild (0–10/cm^2^), moderate (10–100/cm^2^), and considerable (> 100/cm^2^) macrophage infiltration. Subsequently, a consensus score between the independent researchers was determined. An acceptable consensus score was predefined as 60%. It was predefined that all samples would be re-assessed with a senior pathologist if consensus was less than 60%.

### MRI protocol and image evaluation of the Sciatica Trial

MRI scans were performed at baseline by a 1.5 Tesla scanner, and both sagittal T1- and T2-weighted images of the lumbar spine were obtained. Image evaluation of MC was according to the criteria of Modic et al. [17, 18]. Image evaluation was done according to a predefined protocol (Supplementary Table S1) [9]. Definitions of imaging characteristics were based on the recommendations from the combined task forces of the North American Spine Society, the American Society of Spine Radiology, and the American Society of Neuroradiology for classification of lumbar disc pathology. MRIs were evaluated by 2 neuroradiologists and 1 neurosurgeon. All three were blinded to histological data and clinical information. The readers were not involved in the selection or treatment of the patients included. Inter-observer agreement analyses was published earlier by el Barzouhi et al. [6, 7]. For the statistical analyses, the majority opinion of the three independent researchers (answer by at least two of the three MRI assessors) was used.

Type of disc herniation as quantified by MRI was categorized as follows: bulging (or protrusion), extrusion, or not applicable, based on characteristics described by Fardon and Milette [8]. The definition of protrusion as defined by the protocol is more commonly known as bulging in daily clinical practice. Therefore, this term in used in this article. The size of the herniated disc was measured as the surface in squared millimeter. The decrease in size of the disc herniation between baseline and 1 year after randomization was measured as a percentage reduction in surface. MC were scored as type 1, type 2, or type 3 according to Modic et al. [17, 18]. The inter-observer agreement was substantial for the MC (69–97%), as described by el Barzouhi (2014) [7], no kappa calculated because the prevalence of MC types 1 and 3 were too low. At last, inter-observer agreement was substantial regarding disc type (kappa = 0.62) [6].

### Statistical analysis

The categorized histological findings were associated with the MRI variables (type and size of the herniated disc, and disc herniation reduction at 1-year follow-up). In addition, histological findings were split into a group with and a group without MC at the herniated disc level, and comparisons with all MRI variables were repeated for these subgroups. The association between the histological findings and disc type and was tested using χ^2^ tests for categorical data. The association between the histological findings and disc size was done using a one-way ANOVA. At last, the association between the histological findings and disc size reduction was assessed using a Kruskal-Wallis test. *p* values of < 0.05 were regarded as significant.

## Results

### Demographics

Of the 180 patients that underwent surgery, 119 patients’ disc samples were preserved and analyzed. Missing samples were due to multiple reasons: samples were either not collected during surgery, got lost after surgery, not preserved properly, or got lost after preservation. A total of 103 of the 119 patients received an MRI at 1-year follow-up. The baseline characteristics age, gender, BMI, and duration of sciatica prior to surgery of the three macrophage infiltration groups were comparable (Table 1).

**Table 1:** Baseline characteristics of the three histologically defined inflammation groups

	Mild (*N* = 48)	Moderate (*N* = 45)	Considerable (*N* = 27)
Age	40.4 ± 9.6	42.1 ± 10.3	43.7 ± 6.6
Male gender	36 (75.0%)	28 (62.2%)	22 (81%)
Body-mass index	26.0 **±** 4.1	25.8 ± 3.5	25.9 ± 3.2
Duration of sciatica in weeks	9.6 ± 1.9	9.7 ± 2.3	8.7 ± 2.1

Values for gender are *n* and (%) or means ± SD. No significant baseline differences were observed between the three inflammation groups

### The histological data analysis

**Fig. 1:**
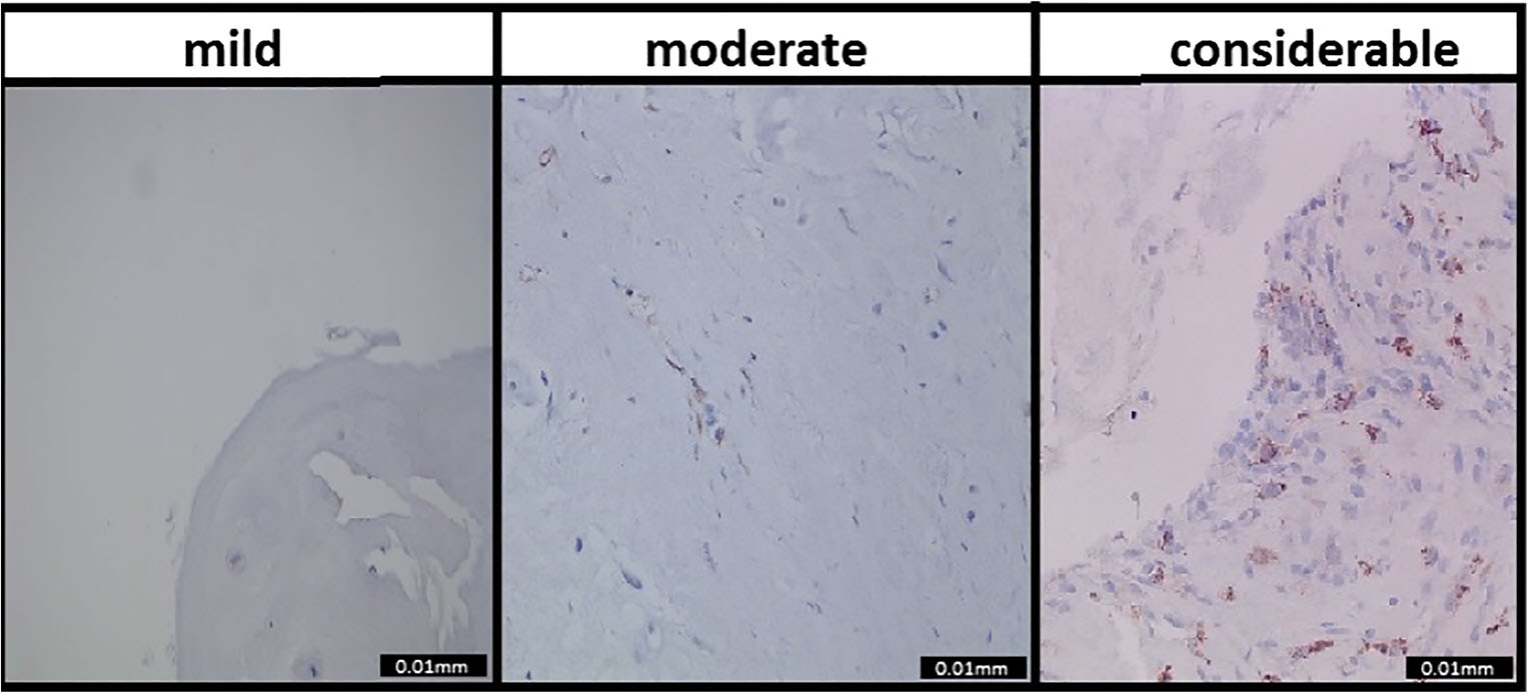
Macrophage infiltration categories. Examples of what was categorized as mild, moderate, and considerable macrophage infiltration

CD68 staining to identify macrophages resulted in the following distribution: 47 (39.5%) patients had mild, 45 (37.8%) patients moderate, and 27 (22.7%) considerable macrophage infiltration (Table 2).The consensus score was excellent (0.96) (Table 2). Examples of the CD68 samples and their categories are shown in Fig. 1.

**Table 2:** Consensus score of the pathological findings. A and B both represent independent observers. The agreement was assessed after the consensus reading

	A vs. B % agreement
Inflammation at baseline (3 categories) • Mild • Moderate • Considerable	95.8 96.8 94.6 96.2

### The MRI data analysis

Forty-one patients demonstrated bulging (protrusion) of the disc, 74 an extrusion, and in 2 patients, discs were scored as not evidently protruding; in 2 cases, information was missing. The disc size was measured in 116 patients with a mean of 77.9 mm^2^ ± 3.6 SE (2 samples were missing). The reduction in disc size was measured over 108 patients, which had a mean of 66.6% ± 4.1 SE, of which 46 disc herniations completely disappeared. Furthermore, a total of 2 patients with MC type 1, 32 patients with MC type 2, no patients with MC type 3, and 85 patients had no MC at the vertebrae adjacent to the herniated disc. Because only two patients showed MC type 1, these samples were excluded from the additional statistical analysis in which only patients with MC were assessed. Thus, in this additional analyses, only MC type 2 were taken into account.

### Associations between number of macrophages and type and size of disc herniation

**Fig. 2:**
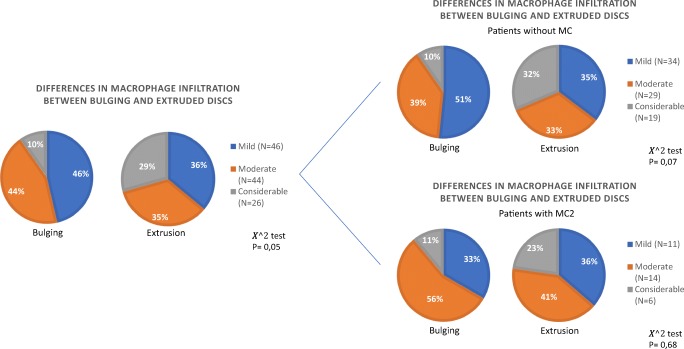
Association between the type of disc herniation and macrophage infiltration at baseline. Pie charts display the distribution of the macrophage infiltration groups in percentages, a χ^2^ test was performed to assess the significance in distribution between bulging and extruded discs, and *p* values are given. Comparison **a** for the whole population, **b** for patients without MC, and **c** for patients with MC

The type of disc herniation at baseline showed a significant association with the degree of macrophage infiltration, suggesting that patients with more macrophage infiltration are more likely to have an extruded disc as compared to a bulging (protruded) disc (*p* = 0.05). The presence of MC type 2 was not of significant influence on this association (no MC: *p* = 0.07; MC type 2: *p* = 0.68) (Fig. 2). In addition, MC2 was not associated with either macrophage infiltration (*p* = 0.53) or type of disc herniation (*p* = 0.32) (Supplementary Figure S1). This verifies that the association between extrusion and macrophage infiltration was not confounded by MC2.

**Fig. 3:**
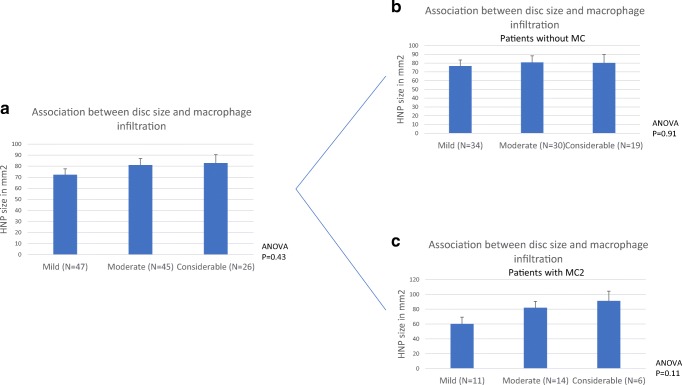
Association between the size of disc herniation and macrophage infiltration at baseline. Macrophage infiltration groups are shown on the *X* axis, values on the *Y* axis are mean HNP surface size in squared millimeter, error bars are SEs and *p* values for the one-way ANOVA test are provided. Comparison **a** for the whole population, **b** for patients without MC, and **c** for patients with MC

Disc herniation size at baseline was not associated with the degree of macrophages infiltration (*p* = 0.43). Again, the presence or absence of MC type 2 changes did not significantly influence this finding (no MC: *p* = 0.91; MC type 2: *p* = 0.11) (Fig. 3).

### Associations between number of macrophages and reduction at 1-year follow-up

The timing of surgery was not equal for all 119 patients. In the sciatica RCT, about 40% of patients that were randomized to conservative treatment crossed over to surgical treatment because of unbearable symptoms. These patients had a longer timespan between start of the complaints and surgical intervention, varying from approximately the same waiting time as the surgical group (mean = 15 days after randomization) to 18 months after randomization. In our sample group, 34 of 119 patients were originally randomized to conservative care and crossed over. Because the follow-up MRI was made at 1-year after randomization and not after surgery, not all of the crossed over patients were comparable with the patients randomized for surgery. Therefore, crossed over patients with waiting time for surgery longer than 6 months were excluded from this analysis, which resulted in exclusion of 7 of the 34 patients.

**Fig. 4:**
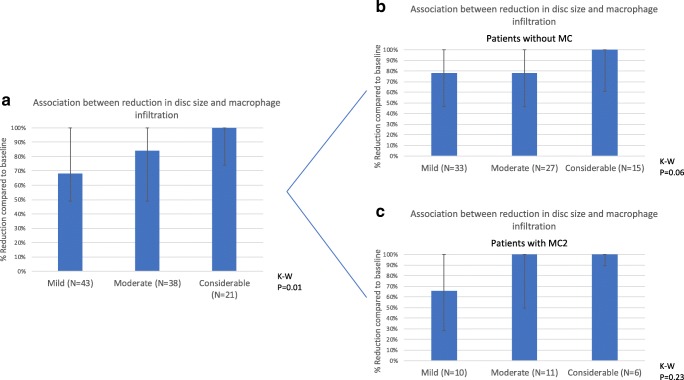
Association between the degree of macrophage infiltration and the percentage surface reduction of the HNP between baseline and 1-year follow-up on MRI. Macrophage infiltration groups are shown on the *X* axis, values on the *Y* axis are median percentages of axial surface reduction compared to baseline, error bars are interquartile ranges and *p* values for the Kruskal-Wallis test are provided. Comparison **a** for the whole population, **b** for patients without MC, and **c** for patients with MC

Reduction of disc herniation, 1 year after surgery, was positively associated with the degree of macrophage infiltration at baseline (*p* = 0.01) (Fig. 4). When testing for the presence and absence of MC type 2 separately, no significant associations were found (no MC: *p* = 0.06; MC type 2: *p* = 0.23; Fig. 4).

In addition, we found that disc extrusion were larger than bulging discs and we found that extruded discs were significantly associated with a larger reduction in size at 1 year (*p<0.01*). Since macrophage infiltration, disc extrusion and relative reduction in size at 1-year follow-up showed multicollinearity; the association between macrophage infiltration and relative reduction at 1 year could be confounded by extrusion, for example, because extruded discs could be easier to resect during surgery. In order to falsify this possibility, an additional analysis was performed with the data from the conservative group of the Sciatica Trial [5]. If this was due to surgery, extrusion would not associate with reduction in the conservative group: The additional analysis showed that extrusion in the conservative group also significantly associated with size reduction at follow-up in the quantitative analyses (Kruskal-Wallis: *p* value = 0.03). The additional analyses are shown in Supplementary Figure S2.

## Discussion

The most important finding of this study is the association between the number of macrophages present at baseline and the decrease in size of the disc herniation at 1-year follow-up in patients with sciatica due to a lumbar disc herniation. This is accompanied by an association between a higher number of macrophages, in extruded as compared with bulging disc herniations. Since extrusion of disc material was demonstrated to resorb faster, patients with a disc extrusion rather than a bulging disc are more likely to benefit from prolonged conservative treatment.

The association between macrophage infiltration and disc extrusion was in line with previous findings of Lohr et al. [14]. This could be explained by the likelihood that disc extrusion as compared with protrusion leads to a larger exposure to systemic circulation, which makes it easier for macrophages to infiltrate the disc during the foreign-body reaction [13]. When performing a separate analysis for patients with and without MC, an MRI feature that has been shown to interact with disc inflammation in recent literature [18], no significant differences were found. However, only in patients without MC an almost significant association between macrophage infiltration and disc extrusion (*p* = 0.07) was demonstrated, while in patients with MC, no such trend was seen (*p* = 0.68). This lack of significance could be explained by the small sample sizes. Alternatively, this finding could indicate that MC type 2 interfere with the normal course of macrophage infiltration, possibly through a disruption in neovascularization [12, 26]. Nevertheless, due to the small sample sizes, these findings should be interpreted with caution.

Furthermore, even though the size of the disc herniation was not associated with the degree of macrophage infiltration at baseline, we did find that the number of infiltrated macrophages was associated with a larger regression of herniated disc material at 1-year follow-up. This indicates the importance of a resorption process induced by macrophages in cleaning up the herniated disc material [13, 21]. Since this study was the first that analyzed this association, no comparison with previous literature could be made.

The additional analyses performed with the data from the conservative group of the Sciatica Trial showed that the association between macrophage infiltration and size reduction of the herniated disc was not confounded by surgery. Hence, we conclude that the reduction was likely to be induced by the infiltrated macrophages.

A different possible confounder on the rate reduction of disc herniation could be the duration of symptoms before surgery. For example, a longer chronic inflammation process could possibly lead to a different result as compared with a short-lived inflammation process [19]. However, since the treatment duration was equal for all macrophage groups, this possibility was excluded.

This study has several strong points. Both the histological and MRI analyses were performed by multiple evaluators and demonstrated substantial inter agreement or consensus scores. Moreover, this study was the first to compare macrophage infiltration with disc parameters on MRI between patients with and without MC type 2. This study also has some limitations. The samples that were evaluated for the histological analysis were old, which might have reduced their quality. Nevertheless, the CD68 staining showed clearly identifiable macrophages and the quality was validated by a senior pathologist. Hence, we assume that the age of the samples did not alter our result. Also, macrophage infiltration does not cover the entire inflammatory picture including the different effects of other inflammatory cells, cytokines, and proteins that also infiltrate the disc as a consequence of neovascularization. However, previous findings have identified macrophages as the main type of inflammatory cells found in discus samples [10, 14]. Thus, it is likely that they contribute the most to the overall inflammation response. Lastly, because all patients already received surgery, it remains unclear whether the reduction of the herniated disc at 1 year is also associated with macrophage infiltration in a population without surgical intervention. Due to ethical considerations, this could not be studied.

In conclusion, macrophage infiltration was positively associated with an extruded type of disc herniation as well as the extent of reduction of the herniated disc during 1-year follow-up in patients with sciatica. In order to evaluate our findings, our results should be repeated by a study with a larger sample size.

## Electronic supplementary material

Table S1 MRI study variables (DOCX 15 kb)

Figure S1 Associations between MC and macrophage infiltration, and between MC and the type of disc herniation at baseline. S1A Pie charts display the distribution of the macrophage infiltration groups in percentages,. S1B Pie charts display the distribution of the bulging and extruded discs in percentages, X^2^ tests were performed to assess the significance in distribution between patients without and with MC, p values are given. (PDF 34.6 kb)

Figure S2 Association between disc type and percentage reduction in axial surface at one year. S2A, compares disc size in the surgical group: disc type is shown on the X axis and values on the Y axis are mean size at baseline, error bars are SE. S2B compares axial surface reduction in the surgical group: disc type is shown on the X axis and values on the Y axis are median percentages of axial surface reduction compared to baseline, error bars are interquartile ranges. S2C compares axial surface reduction in the conservative group: disc type is shown on the X axis and values on the Y axis are percentages of axial surface reduction compared to baseline error bars are SE’s. p values for T-tests or Mann Whitney-U tests are provided accordingly. (PPTX 61.6 kb)
