# Supplementary material for: Lumbar disc extrusions reduce faster than bulging discs due to an active role of macrophages in sciatica
Source: Acta Neurochir (Wien). 2019 Dec 4;162(1):79–85. doi: 10.1007/s00701-019-04117-7 (PMC6942010; doi:10.1007/s00701-019-04117-7)
Supplement: Supplementary file 1 — MRI study variables (DOCX 15 kb) [file 701_2019_4117_MOESM1_ESM.docx]

Supplementary Table S1

**MRI study variables**

| **Disc level** | **Variable** | **Category** |
| --- | --- | --- |
| Disc level with the most severe nerve root compression | Disc level | 1. Not applicable: no nerve root compression  2. L2L3 3. L3L4  4. L4L5 5. L5S1 |
|  | Disc contour at this level | 1. Bulging: presence of disc tissue circumferentially (50-100%) beyond the edges of the ring apophyses  2. Herniation: localized displacement of disc material beyond the normal margins of the intervertebral disc space |
|  | Certainty about the presence of disc herniation | 1. Definite about the presence: no doubt about the presence  2. Probable about the presence: some doubt but probability > 50%  3. Possible about the presence: reason to consider but probability < 50%  4. Definite about the absence: no doubt about the absence |
|  | Loss of disc height at this level | 1. Yes 2. No |
|  | Signal intensity of nucleus pulposus on T2 images at this level | 1. Hypointensity  2. Normal  3. Hyperintensity |
|  | Certainty about the presence of nerve root compression | 1. Definite about the presence: no doubt about the presence  2. Probable about the presence: some doubt but probability > 50%  3. Possible about the presence: reason to consider but probability < 50%  4. Definite about the absence: no doubt about the absence |
|  | Spinal canal stenosis | 1. Yes 2. No |
|  | Disappearance of epidural fat | 1. Completely disappeared  2. Partly disappeared 3. No disappearance |
|  | Presence of vertebral end plate changes and its extent | 1. No VESC (Vertebral Endplate Signal Changes)  2. VESC type 1: hypointense on T1-weighted sequences and hyperintense on T2-weighted sequences  3. VESC type 2: increased signal on T1 weighted sequences and isointense or slightly hyperintense signal on T2 weighted sequences  4. VESC type 3: hypointense both on T1- and T2-weighted sequences  5. VESC type 1 and 2 |
|  | Presence of impaired discs at more than one level | 1. Yes 2. No |
| If a disc herniation is considered | Location | 1. Central zone: zone within the vertebral canal between sagittal planes through the medial edges of each facet  2. Sub-articular zone: zone, within the vertebral canal, sagittally between the plane of the medial edges of the pedicles and the plane of the medial edges of the facets, and coronally between the planes of the posterior surfaces of the vertebral bodies and the under anterior surfaces of the superior facets.  3. Foraminal zone: zone between planes passing through the medial and lateral edges of the pedicles  4. Extra-foraminal zone: the zone beyond the sagittal plane of the lateral edges of the pedicles, having no well-defined lateral border |
|  | Side | 1. Right 2. Left 3. Right and left |
|  | Size disc herniation in relation to spinal canal | 1. Large stenosing, size >75% of the spinal canal  2. Large, size 50-75% of the spinal canal  3. Average, size 25-50% of the spinal canal  4. Small, size <25% of the spinal canal |
|  | Form disc herniation | 1. Protrusion: localized displacement of disc material beyond the intervertebral disc space, with the base against the disc of origin broader than any other dimension of the protrusion.  2. Extrusion: localized displacement of disc material beyond the intervertebral disc space, with the base against the disc of origin narrower than any one distance between the edges of the disc material beyond the disc space measured in the same plane, or when no continuity exists between the disc material beyond the disc space and that within the disc space. |
