# Supplementary figures and images for: Lumbar disc extrusions reduce faster than bulging discs due to an active role of macrophages in sciatica
Source: Acta Neurochir (Wien). 2019 Dec 4;162(1):79–85. doi: 10.1007/s00701-019-04117-7 (PMC6942010; doi:10.1007/s00701-019-04117-7)

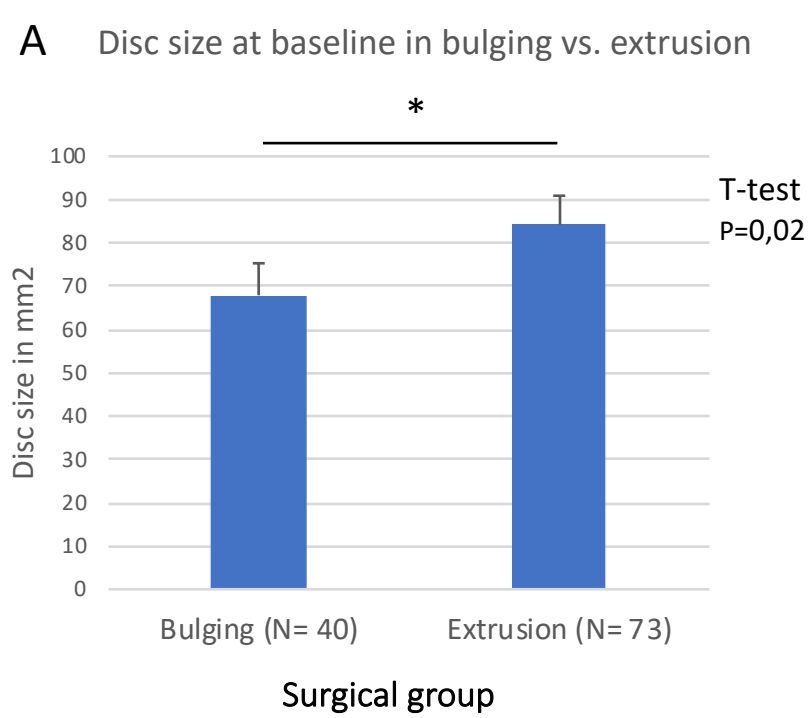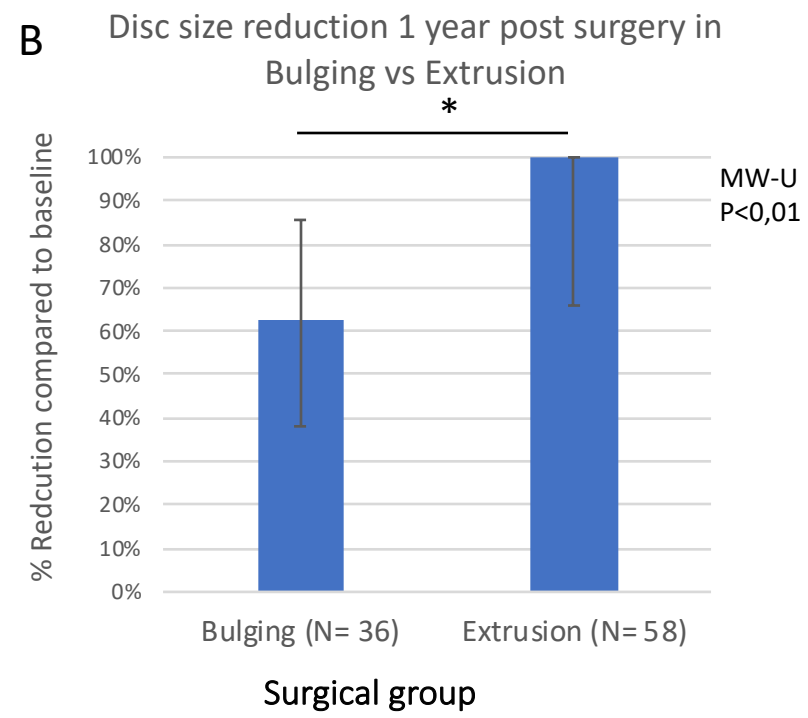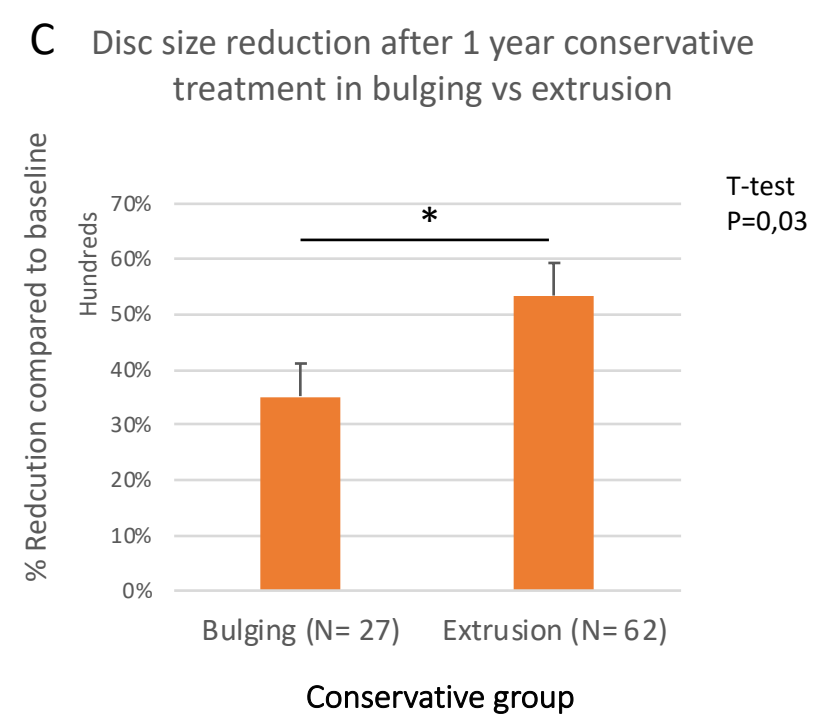

Supplement: Supplementary file 2 — Associations between MC and macrophage infiltration, and between MC and the type of disc herniation at baseline. S1A Pie charts display the distribution of the macrophage infiltration groups in percentages,. S1B Pie charts display the distribution of the bulging and extruded discs in percentages, X2 tests were performed to assess the significance in distribution between patients without and with MC, p values are given. (PDF 34.6 kb) [file 701_2019_4117_MOESM2_ESM.pdf]
