# Supplementary material for: Lumbar disc extrusions reduce faster than bulging discs due to an active role of macrophages in sciatica
Source: Acta Neurochir (Wien). 2019 Dec 4;162(1):79–85. doi: 10.1007/s00701-019-04117-7 (PMC6942010; doi:10.1007/s00701-019-04117-7)
Supplement: Supplementary file 3 — Association between disc type and percentage reduction in axial surface at one year. S2A, compares disc size in the surgical group: disc type is shown on the X axis and values on the Y axis are mean size at baseline, error bars are SE. S2B compares axial surface reduction in the surgical group: disc type is shown on the X axis and values on the Y axis are median percentages of axial surface reduction compared to baseline, error bars are interquartile ranges. S2C compares axial surface reduction in the conservative group: disc type is shown on the X axis and values on the Y axis are percentages of axial surface reduction compared to baseline error bars are SE’s. p values for T-tests or Mann Whitney-U tests are provided accordingly. (PPTX 61.6 kb) [file 701_2019_4117_MOESM3_ESM.pptx]

## Slide 1
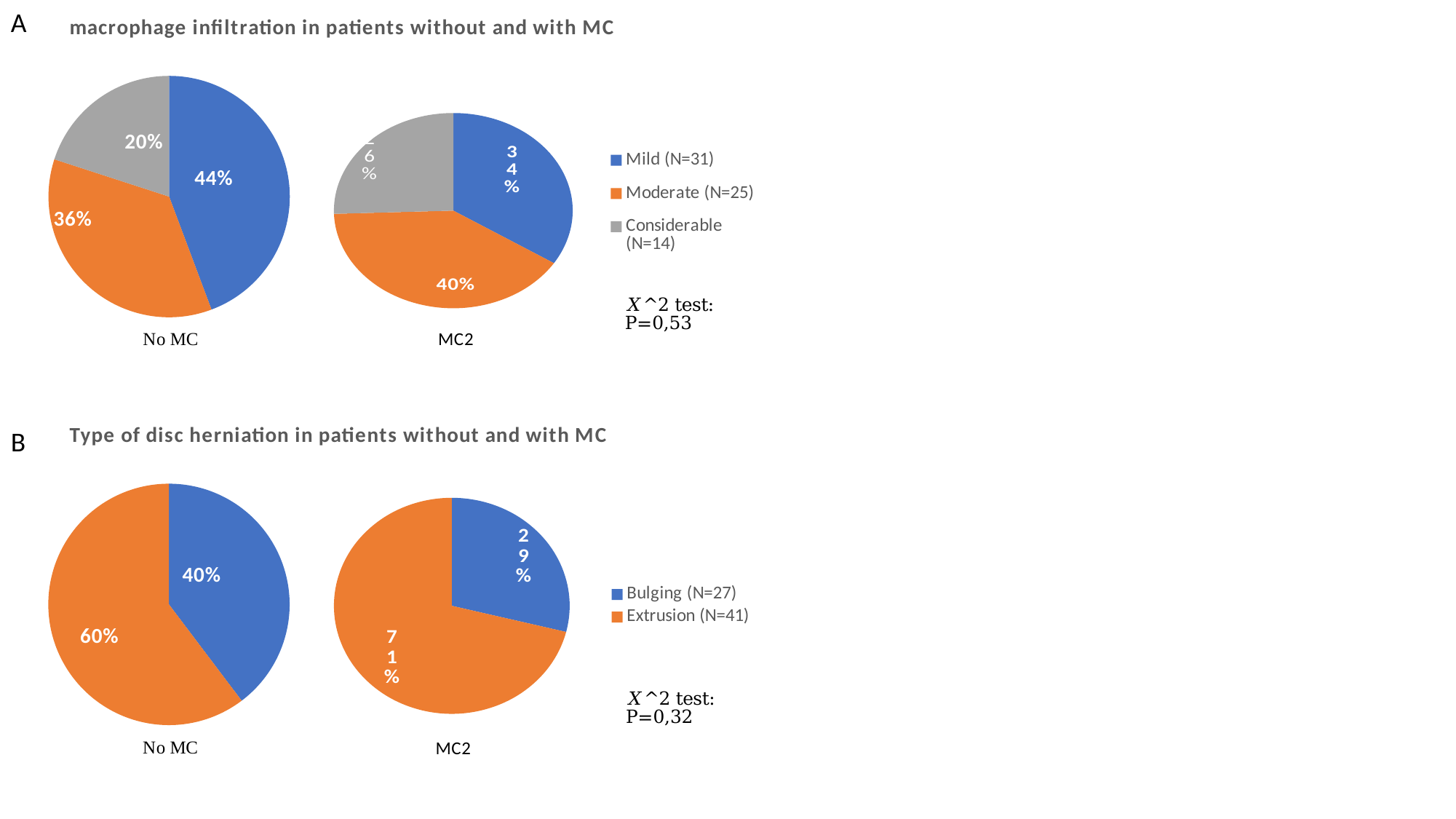

A
### Chart: macrophage infiltration in patients without and with MC
| Category | | |
|---|---|---|
| Mild (N=31) | 31.0 | 16.0 |
| Moderate (N=25) | 25.0 | 19.0 |
| Considerable (N=14) | 14.0 | 12.0 |
### Chart
| Category | |
|---|---|
| Mild (N=16) | 16.0 |
| Moderate (N=19) | 19.0 |
| Considerable (N=12) | 12.0 |
### Chart: Type of disc herniation in patients without and with MC
| Category | |
|---|---|
| Bulging (N=27) | 27.0 |
| Extrusion (N=41) | 41.0 |B
### Chart
| Category | |
|---|---|
| Bulging (N=27) | 13.0 |
| Extrusion (N=41) | 32.0 |
